# Local and Systemic Changes in Lipid Profile as Potential Biomarkers for Canine Atopic Dermatitis

**DOI:** 10.3390/metabo11100670

**Published:** 2021-09-30

**Authors:** Jackeline Franco, Bartek Rajwa, Paulo Gomes, Harm HogenEsch

**Affiliations:** 1 Department of Comparative Pathobiology, Purdue University, West Lafayette, IN 47907, USA; francoj@purdue.edu; 2 Bindley Bioscience Center, Purdue University, West Lafayette, IN 47907, USA; 3 Department of Veterinary Clinical Sciences, Purdue University, West Lafayette, IN 47907, USA; gomesp@purdue.edu; 4 Purdue Institute of Inflammation, Immunology and Infectious Diseases, Purdue University, West Lafayette, IN 47907, USA

**Keywords:** lipidomics, canine atopic dermatitis, biomarkers, diagnostic, disease progression, flow-injection mass-spectrometry, predictive elastic net regression

## Abstract

Lipids play a critical role in the skin as components of the epidermal barrier and as signaling and antimicrobial molecules. Atopic dermatitis in dogs is associated with changes in the lipid composition of the skin, but whether these precede or follow the onset of dermatitis is unclear. We applied rapid lipid-profiling mass spectrometry to skin and blood of 30 control and 30 atopic dogs. Marked differences in lipid profiles were observed between control, nonlesional, and lesional skin. The lipid composition of blood from control and atopic dogs was different, indicating systemic changes in lipid metabolism. Female and male dogs differed in the degree of changes in the skin and blood lipid profiles. Treatment with oclacitinib or lokivetmab ameliorated the skin condition and caused changes in skin and blood lipids. A set of lipid features of the skin was selected as a biomarker that classified samples as control or atopic dermatitis with 95% accuracy, whereas blood lipids discriminated between control and atopic dogs with 90% accuracy. These data suggest that canine atopic dermatitis is a systemic disease and support the use of rapid lipid profiling to identify novel biomarkers.

## 1. Introduction

Atopic dermatitis (AD) is a chronic pruritic inflammatory disease with a strong genetic predisposition. It is the most common inflammatory skin disease in people and dogs, with 4.9 to 7.3% of the adult human population and up to 15% of dogs affected in the United States [1,2,3]. Canine AD (CAD) is a progressive disease that affects the dog’s quality of life and can be immensely frustrating for dog owners [3,4,5]. The diagnosis of CAD is a lengthy process of exclusion based on two sets of criteria with sensitivity and specificity ranging from 79% to 85% [6,7]. To date, there is not an accurate biomarker to discriminate atopic dogs from healthy animals.

The clinical features and pathogenesis of CAD and atopic dermatitis in humans appear to be very similar [8,9]. The disease is thought to be caused by an aberrant immune response to environmental allergens, such as house dust-mite antigens and pollen that penetrate the epidermal barrier [10,11]. The physical barrier is formed by the outermost layer of the skin, the stratum corneum, which consists of anucleated flattened keratinocytes (corneocytes) that are embedded in a lamellar lipid matrix in a “brick-and-mortar” configuration [12,13]. The main classes of lipids that make up the epidermal lipid matrix are free fatty acids (FFAs), cholesterol esters, and ceramides. In addition to their critical function in the skin barrier, lipids also play essential roles as signaling molecules in inflammation and wound healing and have antimicrobial activity by enhancing the effect of antimicrobial peptides [14,15]. CAD is associated with an impaired barrier function, but whether this is a primary defect or secondary to cytokines released during the inflammation is unclear. A limited number of studies have reported changes in the stratum corneum or epidermal lipid composition in CAD, including a reduction in the relative amount of ceramides and variable results for FFA and cholesterol [16,17,18,19,20]. The inconclusive results can be attributed in part to differences in breeds, sample collection, and methodology for lipid analysis.

Recent evidence indicates that AD in people and in dogs is not a single disease but rather a complex syndrome composed of multiple endotypes, with different molecular pathways leading to a similar clinical phenotype [21,22]. As treatments are being developed that target specific molecular pathways, it becomes increasingly important to develop methods to identify these endotypes. Biomarkers are critical tools for the stratification of CAD into subcategories that require individualized treatment regimens. Assessment of disease progression of CAD is challenging and time-consuming as it requires evaluation scores that introduce subjectivity and make comparison among studies difficult [23]. Identifying biomarkers that correlate with the presence of disease and disease severity is crucial for determining the response to treatment in an objective manner. Finally, research into potential biomarkers will likely provide further insight into the pathogenesis of the disease.

Lipidomics has emerged as a valuable tool for biomarker discovery in dermatology [24]. However, the highly demanding analytical techniques used in lipid analysis hinder their use as a routine practice that supports clinicians in diagnosing and assessing treatment response. Therefore, the development of a rapid method that allows repeated testing before and during treatment to evaluate the changes in skin lipid composition and correlations with severity score is needed. While previous reports of lipid changes in CAD used targeted approaches based on previous knowledge, we recently described the development and utility of a nontargeted lipidomics approach, called multiple reaction monitoring (MRM) profiling, to investigate changes in the epidermal lipid composition in a mouse model of atopic dermatitis [25,26]. This method allows for rapid identification of a sample’s lipid fingerprint in a high-throughput manner with minimal sample preparation and a straightforward data-processing workflow. Here, we compiled a method to screen skin swabs collected from lesional and nonlesional skin of CAD patients and skin of control dogs.

Atopic dermatitis in human patients is frequently associated with rhinitis and asthma [27], but there has been growing recognition of an increased frequency of other comorbidities, including cardiovascular disease and autoimmune diseases, suggesting that AD should be considered a systemic disease [28,29]. This prompted us to investigate lipids not only in the skin but also in the blood. Furthermore, we report here the effect of two commonly used pharmacologic treatments for CAD on the skin and blood lipid profiles.

## 2. Results

### 2.1. Dogs and Clinical Response to Treatment

The dogs varied in age from 1 to 11 years. There was no significant difference in age or sex distribution between the control and atopic dogs (Table 1).

**Figure 1:**
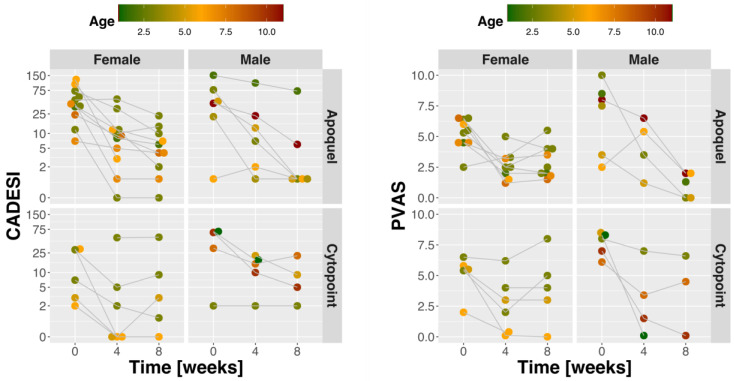
Changes in CADESI and PVAS scores during treatment.

Twenty-two purebred dog breeds were represented, but mixed breeds made up the largest group. The CADESI-4 score is a method to assess the extent and severity of skin lesions in dogs with atopic dermatitis on a scale of 0 to 180. The CADESI-4 score of the atopic dogs enrolled in the study ranged from 1 to 152 with an average of 43.5, and a standard deviation of 38.4 (median 35.5, mean absolute deviation (MAD) = 38.5), which indicates that most dogs had mild to moderately severe atopic dermatitis. The Pruritus Visual Analog Scale (PVAS) ranged from 2 to 10 with an average of 5.9 and a standard deviation of 2. CADESI-4 score and PVAS were correlated at the baseline (*p*-value = 0.04), but there was no significant correlation after treatment. Twenty-seven dogs were treated with either oclacitinib, a Janus Kinase (JAK) inhibitor that primarily targets JAK1-dependent signaling pathways (*n* = 17), or lokivetmab, a caninized monoclonal antibody that targets interleukin-31, a cytokine that induces pruritus in dogs (*n* = 10). CADESI-4 measurements were available at 0, 4, and 8 weeks of treatment for 15 dogs treated with oclacitinib and 8 dogs treated with lokivetmab (Figure 1). Overall, the treatments resulted in substantial (standardized mean difference SMD = 2.17) and statistically significant change in CADESI-4 score (*p* < 0.001). There was only a modest (SMD = 0.39) and not significant (*p* = 0.35) further decline of the CADESI-4 score from 4 to 8 weeks after the initiation of treatment. Similarly, the PVAS score changed significantly after treatment (SMD = 1.68, *p*-value < 0.001). However, there was no observable change from 4 to 8 weeks (SMD = 0.035, *p*-value = 0.99).

### 2.2. MRM Profiling of Skin Samples

**Figure 2:**
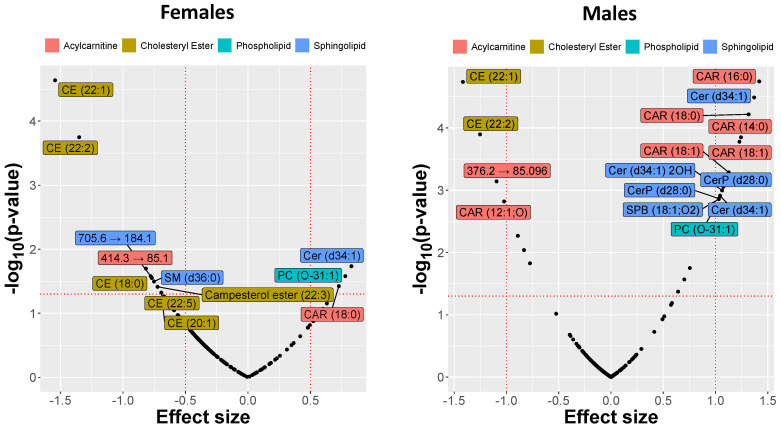
Effect size of changes in lipid abundances in the skin of atopic dogs at baseline vs. healthy controls separated by sex. The effect sizes are represented as standardized mean difference (SMD or Cohen’s *d*).

Mass spectrometry analysis of the skin-swab samples demonstrated significant differences in lipid abundances between controls and atopic dogs at t_0_. Among phospholipids, a decrease was observed in diacylglycerophosphates (PAs), diacylglycerophosphoethanolamines (PEs), diacylglycerophosphoinositols (PIs), and diacylglycerophosphoserines (PSs), but species of the diacylglycerophosphocholine (PC) subclass, including PC (O-31:1), were increased in atopic skin. The sphingolipids had a similarly mixed response with the increased relative abundance of most species, including Cer (d34:1) 2OH and Cer (d34:1), but a decrease for others such as SM (d42:1) and Cer (d44:1). This allowed us to rank and visualize the lipids by the observed effect sizes expressed by SMD (Figure 2).

**Figure 3:**
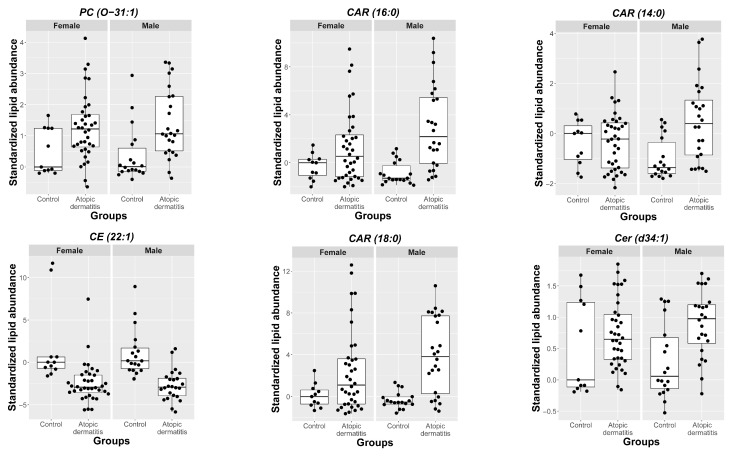
Representative lipids extracted from the skin (before treatment), demonstrating a high level of differences in abundance. The data are standardized to the mean and variance of abundance measured in control samples obtained from female dogs.

The amounts of acylcarnitine CAR(18:0), ceramide Cer(d34:1), and glycerophospholipid PC(O-31:1) were increased in lesional skin from male and female atopic dogs at t_0_ compared to controls, while a substantial decrease in the relative abundances of cholesteryl esters CE(22:1) and CE(22:2) was observed. However, the relative abundance of other lipids such as sphingosine (SPB(18:1;O2)), CerP(28:0), Cer(d34:1)2OH, CAR(16:0), and CAR(18:1) was increased only in atopic males and not females. Comparison between CAD and control groups revealed that the number of different lipids and the effect sizes were overall larger in males than in females (Figure 2). The differences observed in some representative lipids (selected on the basis of the exhibited effect sizes) are illustrated in Figure 3.

**Figure 4:**
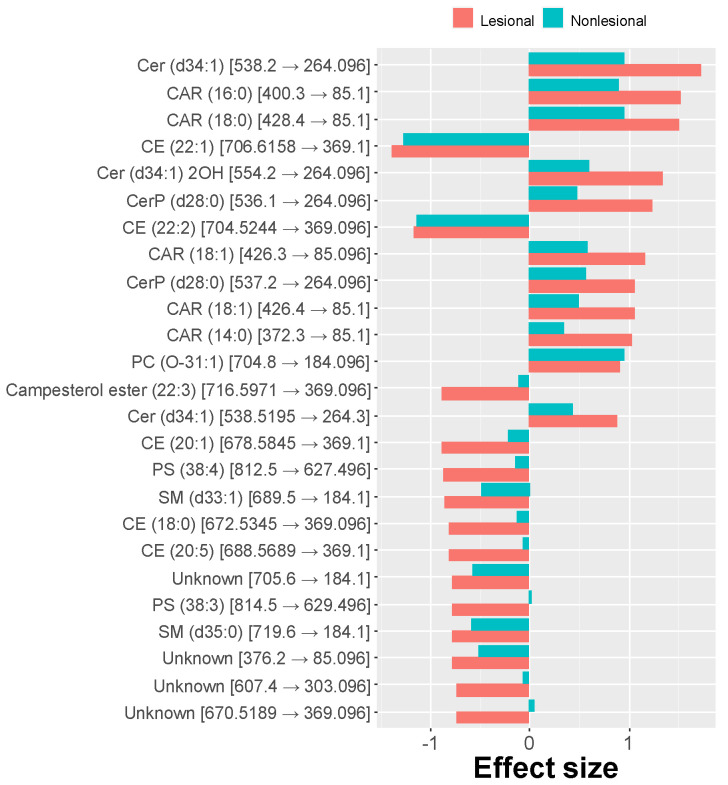
Comparison of effect sizes (standardized mean difference; top 25 absolute values) observed between lipid abundances present in swabs collected from CAD (lesional and nonlesional skin) vs. healthy dogs. The nonlesional skin is affected to a lesser extent than lesional skin.

While comparing effects in lesional and nonlesional skin samples from atopic dogs vs. controls, similar alterations were observed, differing only in the degree of the changes. Again, long-chain acylcarnitines were more abundant in nonlesional skin compared to controls, and the amount of long-chain cholesteryl esters was reduced. Glycerophospholipids were generally increased in nonlesional skin compared to controls, but, as in lesional skin, only PC(O-31:1) was significantly different. The relative abundance of sphingolipids in nonlesional skin was decreased similar to other sphingolipids such as ceramides and ceramides phosphates except for an increase of sphingosine (SPB(18:1;O2)). However, only the decrease of the relative amount of Cer(d34:1) was significant. Some highly predictive lipids were also significantly different in abundance when comparing pairwise lesional and nonlesional skin samples from atopic dogs. For instance, the relative abundance of long-chain acylcarnitines was higher in lesional than in nonlesional skin, while several cholesteryl esters were reduced. The differences between the effect sizes exhibited in lesional and nonlesional skin are presented in Figure 4.

**Figure 5:**
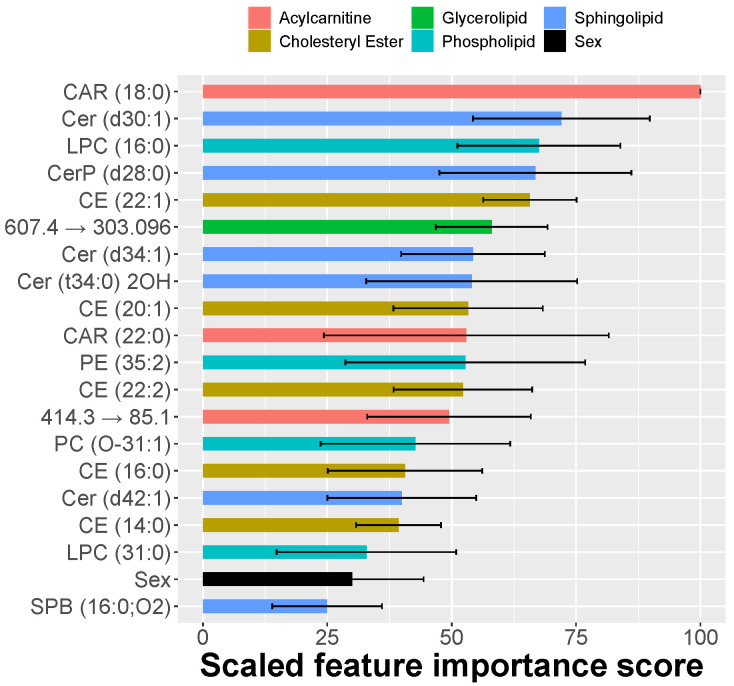
The most predictive skin lipid features for CAD identified by elastic-net-regularized logistic regression. The features include the animals’ sex, underscoring the importance of sex difference in CAD occurrence and diagnosis. The feature importance is expressed as the scale version of the absolute value of the coefficients in the tuned elastic-net model. The training of the model was repeated ten times, and the whiskers illustrate the observed standard deviations.

The profound differences in lipid composition allowed the training of an elastic net logistic classifier (ENET), which achieved a high accuracy (AUC = 0.95; CI = 0.91–0.99). The accuracy of the classifier could be treated indirectly as a measure of features’ quality and their association with the predicted outcome. In contrast to black-box classifiers, such as neural networks, the ante-hoc explainable ENET set-up allowed us to determine the list of the most predictive features. The ENET model identified the combination of CAR(18:0), Cer(d30:1), LPC(16:0), and CerP(d28:0) as the most predictive lipids (Figure 5). Interestingly, some of the lipid features that highly contributed to classification accuracy in the ENET were not recognized by the univariate feature ranking, suggesting that the disease is linked to a complex interplay between multiple lipids and a nonlinear relationship between lipid abundance and the disease manifestation. Including the sex of the animal in the classifier improved the accuracy of the classifier, which further highlights its role in this disease.

**Figure 6:**
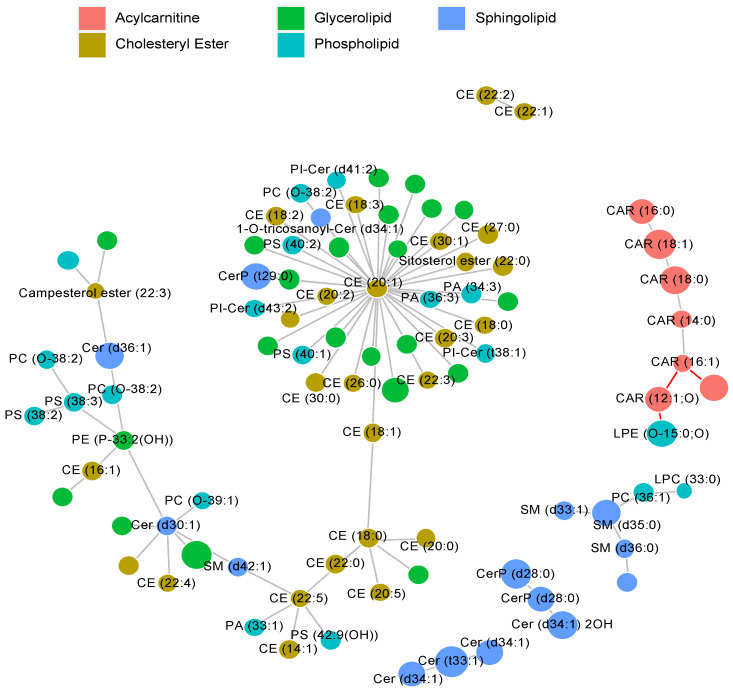
Correlation network demonstrating interdependence between lipid abundances measured in skin samples. The size of the circles illustrates the effect size (difference between the abundances observed in diseased animals and the healthy controls), whereas the edge lengths represent the strength of the correlation. The red edges denote a negative correlation.

We also visualized the interdependence between lipid abundances measured in the skin samples via a correlation network. In order to simplify and declutter the graph, the nodes of the network representing the measured lipids were limited to those lipids which exhibited a substantial difference in abundance between the control and CAD groups (Figure 6).

The data collected from the skin, especially from the lesional areas, are noisy, as many confounding factors, such as mechanical trauma and microbial infections, can affect the sample composition. To determine whether similar changes in lipid composition can be identified in blood as evidence of systemic dysregulation of lipid metabolism, we analyzed dried blood spot (DBS) samples from atopic and control dogs.

### 2.3. MRM Profiling of Blood Samples

**Figure 7:**
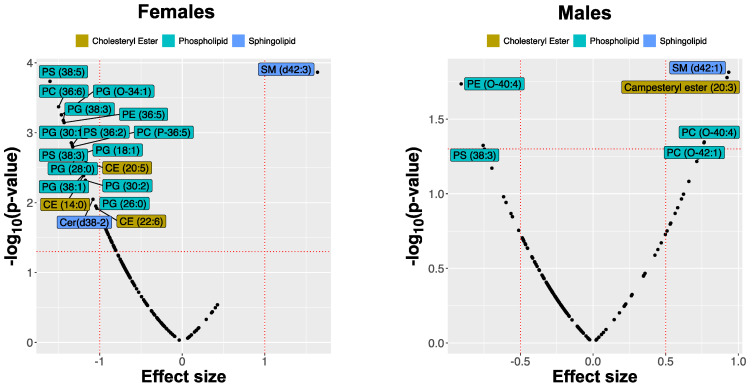
Effect sizes of changes in lipid abundances in the blood of atopic dogs at baseline vs. healthy controls, separated by sex. The effect sizes are represented as standardized mean difference (SMD or Cohen’s *d*).

We observed significant differences in the lipid composition of DBS between atopic and healthy control dogs with sexual dimorphism in the scale of responses. However, in contrast to the skin, female dogs showed more prominent differences (expressed by the absolute value of SMD >1) in some of the lipids. Furthermore, the changes in lipid abundance of certain lipid classes were different from those in the skin. For example, no differences were found among acylcarnitines. Among cholesteryl esters, CE (14:0) and CE (20:5) were substantially lower in abundance in female AD dogs but not in males. Most screened glycerophospholipids and ceramides were reduced in all AD dogs, but only PS(38:5), PS(38:3), PS(36:2), PG(24:0), PG(26:0), PG(28:0), PG(30:1), and PE(36:5), were significantly reduced in blood samples collected from AD female dogs at t_0_, whereas sphingolipid SM(d42:3) was more abundant. In male dogs, the effect sizes were much smaller; however, another sphingolipid SM(d42:1) was significantly increased. The triacylglycerides showed a general decrease in abundance in the CAD group compared with healthy controls. Still, none of these changes were very substantial, and univariate feature ranking did not highlight any of these lipid species. The differences in lipid abundances between the control group and the AD dogs are illustrated in Figure 7.

**Figure 8:**
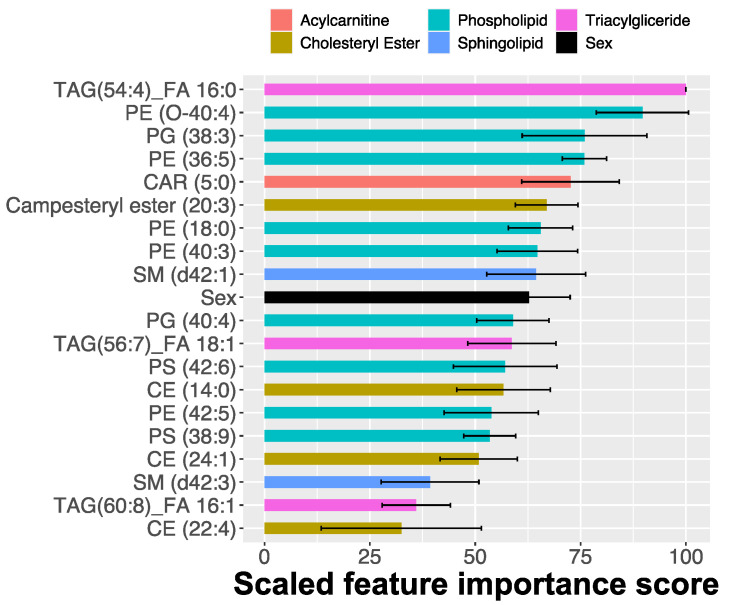
The most predictive blood lipid features identified by elastic-net-regularized logistic regression for CAD occurrence.

As in the case of skin samples, a lipid fingerprint of blood can be used to build a very accurate classifier (AUC = 0.9, CI = 0.82–0.98). The ENET also provided a selection of features contributing to the prediction success. As before, many features that were not picked up by the univariate ranking were identified. The triacylglyceride TAG(54:4)_FA 16:0 was the most prominent one (Figure 8). Interestingly, this lipid did not change in a statistically significant amount between the healthy and the CAD cohorts. However, its presence in the ENET classifier clearly indicated that the abundance of this triacylglyceride contributed considerably to multivariate separation when combined with other lipid features. In blood, as in skin, including the sex of the animal improved the accuracy of the classifier, having a higher ranking in the feature importance scale among the lipids.

**Figure 9:**
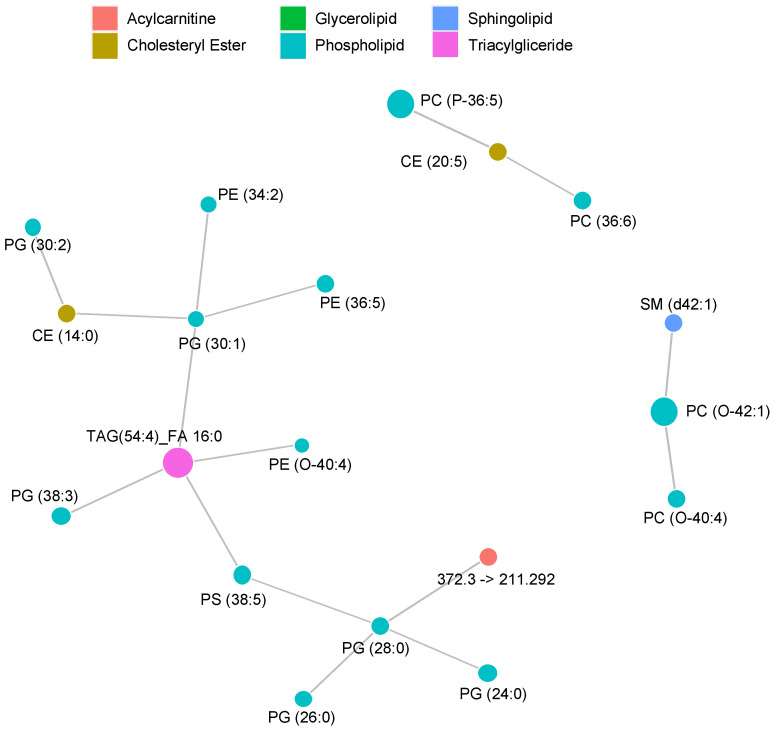
Correlation network demonstrating interdependence between lipid abundances measured in dried blood spot samples.

The visualization of interdependence between abundances illustrates a dramatically less complex network, as the observed effect sizes of CAD in blood samples were much smaller than those shown in skin (Figure 9).

### 2.4. Lipid Changes during Treatment

**Figure 10:**
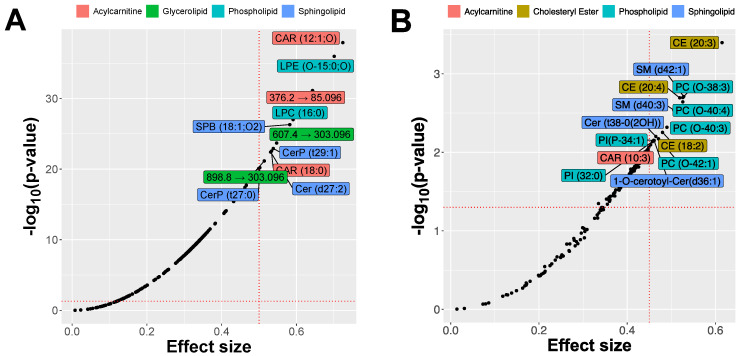
Changes in lipids during treatment identified using linear models; effect size shown as Cohen’s *f*. (**A**): skin swabs, (**B**): dried blood spots.

Treatment caused marked changes in the relative amounts of specific lipids both in skin and in blood. However, the sets of lipids that changed most during the treatment were not the same as those indicative of the disease identified when the comparison between healthy and AD dogs was performed (Figure 10).

**Figure 11:**
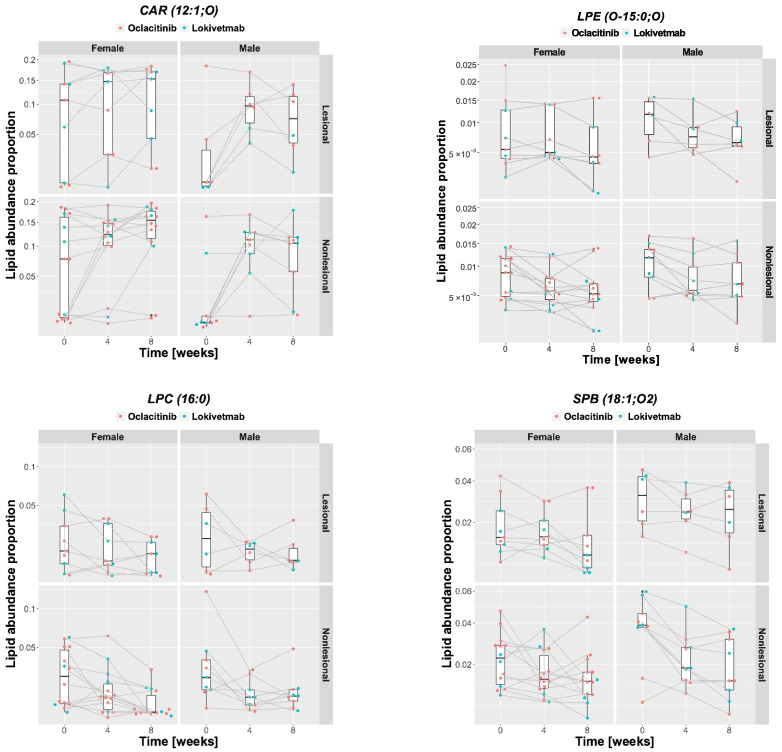
Examples of skin lipid with abundance changing significantly during the treatment.

In skin, many lipids picked up by univariate feature ranking when comparing lesional and nonlesional skin to controls were sensitive to treatment. For instance, the changes in abundance of acylcarnitines, including CAR(18:0) and CAR(16:0), were indicative of treatment progression. There were also significant changes in cholesteryl esters (for example, CE(18:0) or CE(20:1)), glycerophospholipids (e.g., LPC(16:0) or PC(O-31:01)), and sphingolipids (e.g., SPB(18:1;O2), Cer(d34:1)2OH, and CerP(t29:1)). The identification of lipids with the largest effect size in linear models of treatment (Cohen’s *f*) revealed several lipids that were not recognized by previous feature-ranking strategies (for instance, CAR(12:1;O) or LPE(O-15:0;O)). Treatment caused marked changes in the relative amounts of these lipids. Although the patterns seem to be quite complex as the response varies between lesional and nonlesional skin, between males and females, and between treatment regimens, some lipids such as acylcarnitine CAR(12:1;O) tended to increase systematically in relative abundance, whereas other lipids, such as sphingosine (SPB(18:1;O2)), decreased over the course of treatment. Examples of changes in the relative abundance of lipids are shown in Figure 11.

**Figure 12:**
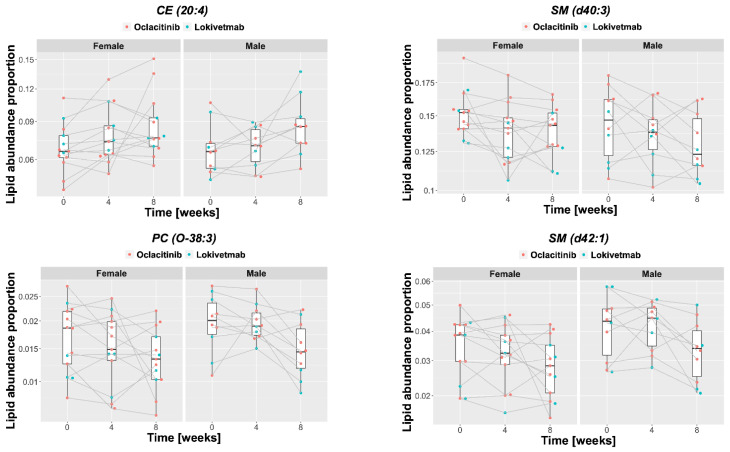
Examples of blood lipid with abundance changing significantly during the treatment.

In blood, the lipid features associated chiefly with changes occurring during treatment were selected via univariate feature ranking based on a set of linear models. Again, several lipids that were not identified in CAD vs. control dog comparison had significant changes, for instance, acylcarnitine CAR(10:3) and several cholesteryl esters (CE(18:2), CE(20:3), CE(20:4)). Some lipids that differed in abundance between AD and healthy controls were also associated with changes during treatment (for instance, SM(d42:1), but not SM(d42:3)). Changes in some triacylglycerides, including TAG(58:2)_FA 16:0, were significant but with modest effect sizes. The alkyl-ether phosphocholines PC(O-38:3), PC(O-40:3), PC(O-40:4), PC(O-42:1), which were identified as increased in AD at t_0_ by the univariate selection, changed significantly over the course of treatment trending toward lower levels in individual dogs. Changes in four lipids over time are shown in Figure 12.

**Figure 13:**
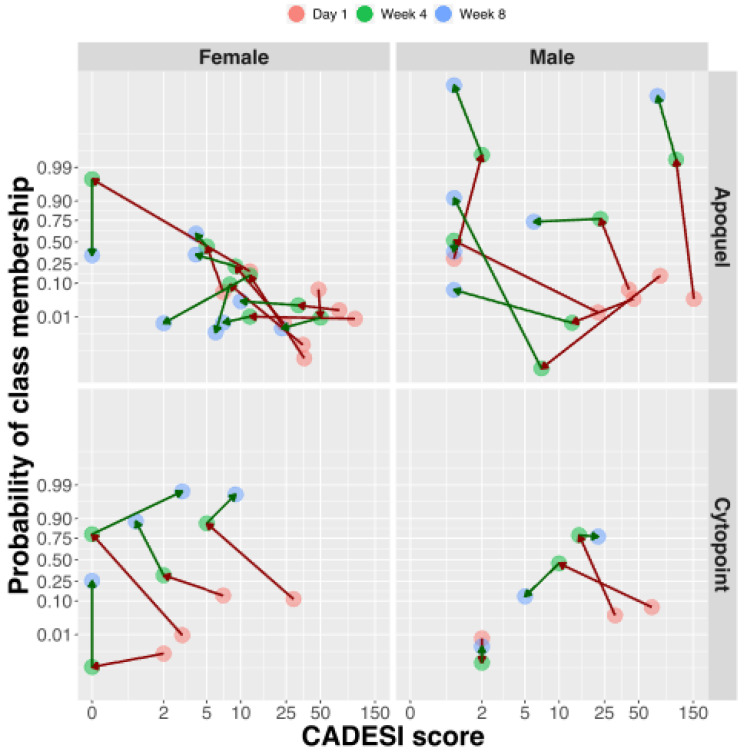
Changes in probability of classifying a fingerprint as healthy vs. the CADESI score. The arrows represent treatment trajectories of individual dogs.

The trajectories of changes in the lipid fingerprint patterns vs. CADESI-4 of individual dogs reflect the response to treatment as the profile classification moves towards the higher probability of healthy phenotype and the CADESI score becomes smaller. This can be seen in Figure 13, where the lipid profiles should migrate towards the upper left corner of the plot to indicate improvement. While most of the dogs indeed show progress during the treatment, the trajectory plot also identifies the animals that are not responding to therapy and those taking longer to display a change in CADESI, despite having their lipid fingerprints modified.

## 3. Discussion

All atopic dogs treated with oclacitinib or lokivetmab had a significantly improved skin condition after 4 weeks of treatment, consistent with previous reports [30,31,32]. The therapeutic response to oclacitinib and lokivetmab appears to be similar, although lokivetmab was more effective than oclacitinib as a preventive treatment in a challenge model of CAD [33]. CADESI-4 and PVAS correlated at the baseline, but that correlation was lost after treatment. The inconsistency of CADESI and PVAS, also reported in other studies [33], highlights the fact that these scoring systems are not robust enough to be used across studies.

In this study, we determined the lipid profile in samples collected with swabs from lesional and nonlesional skin of atopic and control dogs. Previous studies analyzing the skin from dogs with atopic dermatitis have often involved invasive biopsy methods. However, topical extraction with organic solvents or scraping with a surgical blade gave more reproducible results than data collected from epidermis separated from full-thickness skin biopsies using heat [34]. The non-invasive sampling using rayon-tipped swabs allowed for repeated sample collection with minimal patient discomfort and faster sample processing. This procedure yielded sufficient material for lipid extractions and mass-spectrometry analysis. The specimens were analyzed by a direct sample infusion high-throughput lipidomics approach, as previously described for mice [25,26].

The lipid changes in the skin of atopic dogs were notably dependent on sex. We previously reported sexual dimorphism of skin lipids in a mouse model of dermatitis with significant effects on sphingolipids and phospholipids [26]. Here, we found that the relative amounts of lipids were also linked to the sex of the dogs; some lipids, specifically sphingolipids, CEs, and PLs, were affected differently by AD in males and in females. In human patients, epidemiological studies have shown that AD is more prevalent in females after puberty but can be more severe in males, suggesting a role of sex hormones [2,35,36,37]. On the other hand, there appears to be no sexual predisposition in dogs to the development of AD [38,39]. The majority of dogs in our study were neutered and spayed, suggesting that the observed differences between sexes may be attributed not only to sex hormones, but also to genetic differences. To the best of our knowledge, no previous CAD studies have reported an effect of sex on skin lipid composition, and this is a subject that warrants further investigation.

Mass-spectrometry analysis revealed significant differences in lipids from atopic lesional skin compared to controls. There was a significant increase of 34-carbon ceramides and an overall decrease of the detected sphingomyelins in lesional skin samples from the AD dogs compared to controls. Dysregulation of ceramides resulting in impairment of the barrier function has previously been reported in atopic dogs [16,20,40]. While a decrease of ceramides and SM in the stratum corneum of AD dogs may lead to a disorganized lipid matrix [16,41,42], we found that some moieties increased in abundance in CAD skin, for instance, Cer(d34:1). This is in agreement with an increase of shorter non-esterified ceramides in human AD [43,44]. In skin swabs from lesional CAD skin, relative amounts of long-chain acylcarnitines were increased compared to controls. Dysregulation of acylcarnitines has also been reported in human AD and in a mouse model [25,26,45]. Elevated long-chain acylcarnitines can play a proinflammatory role in disease by activating the JNK/ERK/p38 MAPK stress pathways [46] and triggering cellular stress [46,47]. Carnitines are also involved in a dysregulated energy metabolism in AD [48]. Cholesteryl esters were decreased in lesional AD skin compared to healthy dogs. Cholesterol is esterified into CE to be more efficiently transported in lipoproteins to tissue. The CEs act as a storage form of cholesterol and form the majority of sterols in sebaceous glands and hair follicles [49]. Cholesterol is one of the major lipid components of skin and is fundamental to the epidermal lipid barrier function [50], but a previous analysis did not show differences in atopic dogs versus controls [20]. In our mouse model, CEs were identified as one of the most important lipid categories for the classification of disease progression [26].

Glycerophospholipids are fundamental for lipid membrane stability and dynamics. The observed general PL decrease in lesional skin compared to controls may affect cell differentiation, signaling, and antioxidative capacity [51,52,53,54].

Studies of nonlesional skin of atopic dogs have shown ultrastructural differences in the stratum corneum with lower levels of fatty acids and ceramides than healthy dogs that could predispose to an impaired barrier function [55]. When analyzing the relative amounts of lipids in nonlesional skin of atopic dogs, we found that the abundance of five lipid ions was significantly different from controls with an increase of CAR(18:0), PC(O-31:1), and Cer(d34:1), and a decrease of CE(22:1) and CE(22:2). These lipids were also altered in lesional skin, but there was no difference between lesional and nonlesional samples for CE(22:2) and PC(O-31:1), suggesting that their concentrations do not change with disease progression. The differences in lipid composition between the skin of control dogs and nonlesional skin of atopic dogs suggest that a molecular prediction of disease development is feasible. Furthermore, this indicates that the exact location from which skin samples for lipid analysis are collected is not critical, and, in fact, it may be preferable to obtain skin samples from less-affected skin.

Lipidomic analysis of DBSs was done to determine if changes could be identified in the blood lipid profile, which would suggest a systemic nature for the disease. The use of whole blood preserved in DBS cards allows a comprehensive analysis of lipids in both cellular and plasma components of blood. Previous studies suggested no significant differences in fatty acids in serum and plasma samples from control and atopic dogs [56,57]. However, changes in the lipid composition were present in erythrocyte membranes of atopic dogs compared to controls [57]. Ceramides and long-chain carnitines were increased in skin swabs but not in blood samples from the atopic dogs compared with controls, demonstrating that these particular changes were restricted to skin. The increase of SM(d42:3) in females and SM(d42:1) in males highlights the potential role of sphingolipids as bioactive molecules in AD [58]. Cholesteryl esters were decreased in both the skin and blood of AD dogs, although only the changes in the blood of female dogs were significant. Interestingly, PLs were increased in the skin but reduced in blood from AD dogs, and a larger number of different lipid ions were decreased in females than males. The changes in sphingolipids, PLs, and CEs in blood suggest systemic aberrations in lipid metabolism in CAD that are markedly affected by the sex of the dog.

The diagnosis of atopic dermatitis, assessment of the severity, and the response to treatment are based on clinical evaluation of the patient. Several disease-severity scoring systems have been developed for the stratification of human atopic dermatitis patients, but they are often complex and suffer from inter-observer variability, making them unreliable for comparisons across studies [59,60,61]. Several blood molecules have been investigated in human studies as potential biomarkers of atopic dermatitis, including TARC/CCL17, macrophage-derived chemokine, IL-22, pulmonary and activation-regulated chemokine, sIL-2R, soluble E-selectin, C-reactive protein, and IL-16 [62,63]. Similarly, there is no specific diagnostic test for atopic dermatitis in dogs. Diagnostic criteria and scoring systems have been defined and refined by several investigators [6,7,64,65,66], but these remain based on physical evaluation of the patient and exclusion of other causes of pruritus and dermatitis. A few recent studies have investigated changes in concentrations of cytokines and other proteins in blood of dogs with AD as potential biomarkers. Increases in the serum concentration of IL-31 and CCL17 correlated positively with the severity of disease in atopic dogs [67,68]. In contrast, the concentration of C-reactive protein in the blood of atopic dogs after allergen immunotherapy had no clinical use as an indicator for treatment or disease progression [69]. An aim of this study was to identify lipid features from blood and skin samples that are highly predictive of health status. The feature selection combined with classification employing regularized logistic regression demonstrated that abundances of a relatively small number of lipids could predict the health status of the tested animals utilizing either skin or DBS samples. The achieved accuracies (AUC over 0.9) indicate a strong relationship between the measured lipid profiles and the clinical manifestations of CAD. It is important to note that in the case of skin samples, the classifier was successfully trained and cross-validated on data from lesional and nonlesional skin. This supports the notion that the global lipid compositions in lesional and nonlesional skin are similar. Therefore, we may surmise that specific lipids in the skin of animals suffering from AD are systemically altered even though the majority of the skin surface may appear normal and healthy. These results are consistent with previous reports of changes in ceramides in nonlesional skin from atopic dogs [16,18]. Our data also suggest that lipid changes in atopic dogs occur independently of inflammation and trauma from scratching and biting of pruritic skin, although these may further affect the lipid composition. The systemic changes in lipid composition of blood and skin may affect the barrier function of the epidermis and increase the susceptibility of animals to percutaneous sensitization to allergens that lead to inflammation. Cytokines associated with allergic inflammation, such as IL-4, IL-13, IL-31, and TNF-α, can affect lipid synthesis and further diminish the barrier function of the epidermis [70]. In addition, systemic lipid dysregulation may cause changes in keratinocyte differentiation as certain nuclear receptors that are critical for differentiation respond to cellular lipid levels [71,72]. The lipidomics methodology presented here is an objective molecular analysis that can be standardized across studies and facilitate the research of CAD. The results suggest that the method can serve as a tool for the diagnosis of CAD at the early stages of the disease, which may improve the response to treatment. Furthermore, the straightforward sample collection and storage is convenient to perform in clinical practice as it is non-invasive and can be used to evaluate the response to treatment without the need for repetitive biopsies necessary for other types of clinical analysis.

Treatment with either oclacitinib or lokivetmab not only ameliorated the clinical condition of the patient but also shifted the overall lipid fingerprint toward normalcy. Oclacitinib is a Jak1-inhibitor and may affect lipolysis and adipogenesis directly [73,74] or indirectly by blocking signaling through IL-31 and other cytokine receptors. Lokivetmab is a neutralizing monoclonal antibody that targets IL-31 and may prevent the effect of IL-31 on lipid-processing enzymes [70].

It is important to note that the changes in lipid fingerprint composition and the movement of the pattern towards the “healthy” fingerprint do not necessarily correlate with the change in the CADESI score or the pruritus score. Similarly, increased skin hydration after treatment with oclacitinib and lokivetmab did not correlate with the clinical score [33]. Transepidermal water loss and skin hydration have been linked to the ability of the lipid matrix of the stratum corneum to maintain adequate water levels within the epidermis [75,76,77]. This indicates that the lipid fingerprint could be a leading or a lagging indicator of the disease manifestation. The correlation between CADESI and the lipid fingerprint may be affected by the dog’s age, sex, and the prescribed treatment. For the identification of features associated with the effect of treatment, we employed linear mixed models to pick the features mostly affected by treatment time points. Although these strategies are commonly used in systems biology studies, they miss possible interactions between features. Therefore, in addition, we constructed correlation networks demonstrating interdependence between selected features. The network architecture, combined with univariate and multivariate feature selection, allowed us to identify possible candidates for a robust lipid profile that could serve as a joint biomarker of treatment response, guiding the expected improvement a patient should exhibit by a certain time point. The incorporation of such profiles in diagnostic practice could significantly contribute to the elimination of scoring subjectivity.

## 4. Materials and Methods

### 4.1. Animals and Sample Collection

The study was approved by the Purdue Animal Care and Use Committee (PACUC protocol 1510001312). Owners were informed of the goal and scope of the study and signed an informed consent form. Thirty control and thirty dogs with atopic dermatitis were recruited for this study. All dogs were client-owned; they were at least 12 months of age and had overall good health. CAD patients had a documented history of seasonal or non-seasonal pruritus and were diagnosed according to Favrot’s diagnostic criteria [7]. The patients had not received any immunosuppressive or anti-inflammatory treatment for at least two weeks prior to enrolment. At enrollment, the signalment of the dogs was collected along with the Pruritus Visual Analog Score (PVAS) provided by the owners, and the severity of the disease was scored using CADESI-4 during clinical examination [65]. A blood sample was collected by venipuncture, spotted on a card (dried blood spot (DBS); Whatman 903 protein saver card, Sigma-Aldrich, St. Louis, MO, USA), and stored in a sealed plastic bag at −80 °C until processing. Skin samples were collected from lesional and nonlesional skin of dogs with atopic dermatitis and normal skin of the flank of control dogs by vigorously rubbing the skin with rayon-tipped swabs. Nonlesional and control skin samples were collected after carefully trimming the hair with scissors. Swabs were stored in individual plastic containers at −80 °C until processing. Based on their individual clinical assessment, the CAD patients were prescribed either oclacitinib (Apoquel^®^, Zoetis; Kalamazoo, MI, USA) (*n* = 17) or lokivetmab (Cytopoint^®^, Zoetis; Kalamazoo, MI, USA) (*n* = 10). One dog was initially treated with oclacitinib and then changed to lokivetmab, one dog was started with prednisone and subsequently treated with oclacitinib, and one dog was treated topically with shampoo. The dogs returned at 4 and 8 weeks of treatment for follow-up examination and skin and blood collection. Three dogs (two treated with lokivetmab and one with oclacitinib) did not return for the eight-week follow-up visit. The treatment of secondary skin and ear infections with systemic and/or topical antimicrobials was tailored for each patient.

### 4.2. Extraction of Skin Samples

The skin samples were sonicated in an ultrasonic cold bath for 3 min with 200 μL of 70% ethanol and extracted using 790 μL of a chloroform/methanol/water (1:2:0.8) mixture. The combined organic fractions were centrifuged, and the bottom phase was transferred and evaporated. Dried lipid extracts were reconstituted in 40 μL methanol/chloroform at a 3:1 volume ratio and further diluted 5-fold with an injection solvent of acetonitrile/methanol/300mM ammonium acetate (3:6.65:0.35 *v/v*).

### 4.3. Extraction of DBS Samples

The DBS samples were transferred to a 2-mL vial, soaked with 200 μL of ultrapure water for 5 min, and vortexed for 5 min to allow the release of the sample from the paper. Lipids were extracted with 1100 μL of a chloroform/methanol/water (1:2:0.8) mixture and treated as described above. Lipid extracts were solubilized in 40 μL of methanol/chloroform (3:1 *v/v*) and mixed the injection solvent in 1:10 ratio.

### 4.4. MRM Profiling

The extracted samples were analyzed by MRM profiling on a triple quadrupole mass spectrometer equipped with a Jet Stream ESI ion source, as previously described [25,26]. Briefly, mass-spectrometry discovery analysis was performed using neutral loss and precursor ion scans for phosphocholines (PCs), phosphoserines (PSs), phosphoethanolamines (PEs), sphingomyelins (SMs), acylcarnitines (CARs), cholesterol esters (CEs), ceramides (Cers), diverse fatty acid acyl residues, and free fatty acids in the positive and negative mode in composite samples of atopic and healthy-control groups created by pooling aliquots of 5 µL from individual lipid extracts. A total of 222 lipid features from the discovery analysis of skin swabs and 148 lipid features from DBS were identified and compiled in two MRM methods. Each sample was individually screened in a high-throughput manner (approximately 3 min/sample) by injecting 8 µL lipid extract through the micro-autosampler (G1367A) into a QQQ6410 triple quadrupole mass spectrometer (Agilent Technologies, San Jose, CA, USA) equipped with a Jet Stream ESI ion source. The tentative identification of the lipid features was determined using the reference databases Lipidmaps (http://www.lipidmaps.org/, accessed on 22 May 2020) and METLIN (https://metlin.scripps.edu, accessed on 22 May 2020), as well as MS/MS experiments. The lipid features were labeled with their class abbreviation (e.g., PS, PE) followed by the number of carbon atoms in the esterified fatty acid, a colon, and the number of carbon–carbon double bonds in parentheses, such as PS(40:1). Some detected and tentatively attributed lipid ions could be subject of isotopic interferences. The unidentified lipids were labeled with their m/z and corresponding lipid-class fragment. As previously reported, the method has a linearity and dynamic range of four orders of magnitude from 1 to 10,000 ppm [25]. The raw data were deposited in the public mass spectrometry repository MassIVE (MSV000087837).

### 4.5. Statistical Methods

An in-house script was developed and used to obtain the ion intensities of each m/z monitored normalized by the total ion intensity of each sample before statistical analysis. For univariate and multivariate statistical analysis, the normalized relative amounts of these values were transformed using the isometric log-ratio or α-transformation [78,79,80] to correct for the fact that data lie on one-dimensional simplex. To evaluate the importance of different lipids to manifestations of CAD, we assumed that relative lipid abundance, which leads to a more predictive model for the occurrence of CAD and changes in disease manifestation during the course of treatment, is of higher relevance. Therefore, the lipid importance is scored in three different ways: (i) by building multiple univariate models and computing the effect sizes (Cohen’s *d*) expressing the difference between healthy control and untreated animal group for every lipid, (ii) by building a set of linear mixed models and computing effect sizes of changes occurring over the time of treatment (η^2^ or Cohen’s *f*), and (iii) by building a logistic regression classifier with elastic-net regularization, and listing the features selected during the training process as the most contributing to the classifier accuracy [81]. These feature-selection approaches were employed for both the skin-swab and DBS data. The results of the selection are shown as plots in which the *p*-value is indicated on the *y*-axis, and the effect size is shown on the *x*-axis. The effect size could be either negative or positive for the healthy-vs-untreated model, signifying the increase or decrease in the relative abundance of a lipid. The outcome of the elastic-net regressors was illustrated by barplots showing the importance of the variables in terms of the absolute values of the selected coefficients in the sparse elastic-net model. The statistical analysis was performed using R-language for statistical computing.

## 5. Conclusions

In conclusion, we detected global lipid changes in skin and blood of CAD patients and identified fingerprints that can accurately identify atopic dogs using lesional or nonlesional skin as well as DBS. Limitations of our study include the heterogeneity in breed, age, and diet, all of which may affect the skin and blood lipid profile. Nevertheless, the results of this study clearly demonstrate that the lipid abnormalities observed in AD in dogs are not limited to the stratum corneum or the epidermis of inflamed skin but are also present in the blood and nonlesional skin (albeit with smaller effect sizes), suggesting that CAD is a systemic disease with general dysregulation of lipid metabolism. Therefore, the possibility to screen for CAD in dogs using lipid profiling before the clinical onset of the disease exists should be further investigated. Furthermore, the study identified a significant sexual dimorphism in the lipid changes in the skin and blood of dogs with atopic dermatitis. That the lipid fingerprint is responsive to treatment and that intervention improves the clinical outcome suggests that mechanistic studies of the related lipid pathways identified here could be helpful to identify possible treatment targets.

## Figures and Tables

**Table 1:** The mean and median age of the dogs.

Status	Sex	Mean Age	SD	Median Age	MAD
**Atopic**	Female	4.63	1.93	4.00	2.22
	Male	5.27	3.23	5.00	2.97
**Healthy**	Female	6.60	2.99	5.50	2.22
	Male	6.37	3.40	5.00	1.48

## Data Availability

Raw data has been deposited in the public mass spectrometry repository MassIVE (ftp://massive.ucsd.edu/MSV000087837/).
